# Effectiveness of short message services reminder on childhood immunization programme in Kadoma, Zimbabwe - a randomized controlled trial, 2013

**DOI:** 10.1186/s12889-015-1470-6

**Published:** 2015-02-12

**Authors:** Donewell Bangure, Daniel Chirundu, Notion Gombe, Tawanda Marufu, Gibson Mandozana, Mufuta Tshimanga, Lucia Takundwa

**Affiliations:** Department of Community Medicine, University of Zimbabwe, Harare, Zimbabwe; City Health Department, Kadoma City Council, Kadoma City, Zimbabwe

**Keywords:** Randomized control trial, Immunization, Kadoma

## Abstract

**Background:**

Globally, non-attendance for immunization appointments remains a challenge to healthcare providers. A review of the 2011 immunization coverage for Kadoma City, Zimbabwe was 74% for Oral Polio Vaccine (OPV), Pneumococcal and Pentavalent antigens. The immunization coverage was less than 90%, which is the target for Kadoma City. Adoption of short message services (SMS) reminders has been shown to enhance attendance in some medical settings. The study was conducted to determine the effectiveness of SMS reminders on immunization programme for Kadoma City.

**Methods:**

A randomized controlled trial was conducted at Kadoma City clinics in Zimbabwe. Women who delivered and were residents of Kadoma City were recruited into the study. In the intervention group, SMS reminders were sent at 6, 10 and 14 weeks in addition to routine health education. In the non-intervention no SMS reminders were used, however routine health education was offered. Data were collected using interviewer administered questionnaire. Data were analyzed using Epi Info 7™, where frequencies, means, risk ratios and risk differences were generated.

**Results:**

A total of 304 participants were recruited, 152 for the intervention group and 152 for the non-intervention group. The immunization coverage at 6 weeks was 97% in the intervention group and 82% in the non-intervention group (p < 0.001). At 14 weeks immunization coverage was 95% for intervention and 75% for non-intervention group (p < 0.001). Those who did not delay receiving immunization at 14 weeks was 82% for the intervention and 8% for non-intervention group. Median delay for intervention was 0 days (Q_1_ = 0; Q_3_ = 0) and 10 days (Q_1_ = 6; Q_3_ = 17) for non-intervention group. The risk difference (RD) for those who received SMS reminders than those in the non intervention group was 16.3% (95% CI: 12.5-28.0) at 14 weeks.

**Conclusion:**

Immunization coverage in the intervention group was significantly higher than in non-intervention group. Overall increase in immunization coverage can be attributed to use of SMS.

**Trial registration:**

ISRCTN70918594. Registration Date: 28 August 2014.

## Background

Vaccine preventable diseases remain one of the major causes of morbidity, disability and mortality in African Region. Measles and neonatal tetanus in particular account for most of the 11.4 million deaths recorded each year among the under five years of age. The Regional Strategic Plan of World Health Organization for African Region (WHO/AFRO) on immunization calls on countries to strengthen their immunization systems, accelerate diseases control and introduce new vaccines and technological innovations [1,2].

Immunization coverage is the proportion of vaccinated individuals amongst the target population. It is one of the most important indicators of a successful immunization programmes. To achieve sustained and equitable access to good quality immunization services, the Global Alliance for Vaccines and Immunizations (GAVI), proposed Reaching Every District (RED), an approach to be implemented in an integrated manner using immunization as a platform for a range of priority interventions [1-4].

Short message services (SMS) is a text messaging component of phone, web or mobile communication systems using standardized communications protocols that allow the exchange of short text messages. Adoption of short message services has been shown to enhance the attendance in medical setting [5]. In some settings the system may provide a cheap, automated alternative means of communication. Text messaging reminder systems are a cost effective way of improving attendance in a variety of healthcare settings [5-9]. Due to the complicated nature of child immunization and the penetration of mobile phones, text messaging maybe a successful strategy to increase immunizations in some settings [5-13].

Zimbabwe introduced a new immunization schedule in 2012. Newly born babies are now expected to begin vaccinations at 6 weeks instead of the previous three months after the initial vaccine given at birth. This follows introduction of the new vaccination schedule and the pneumococcal conjugate vaccine in July 2012. According to the new vaccination schedule, immunization is now starting with BCG at birth. Other antigens will be administered at six, 10 and 14 weeks instead of three, four and five months. The vaccination schedule now ends with the 18 months booster [4].

Kadoma City is an urban area located in the Mashonaland province of Zimbabwe. The total population is 92, 000 (CSO 2012). In terms of health delivery the city is served by one public hospital (Kadoma General Hospital) and five health centres owned by Kadoma City Council. Kadoma City has an estimated population of 2469 for the under 1 year. It is estimated that Kadoma City has 100% mobile network coverage and at least each household has one functional mobile phone.

A review of the 2011 consolidated monthly return forms (T5) reveals that the annual measles coverage for Kadoma City was 74%. This measles coverage was far below the national and the district target of 90%. The measles dropout rate was 13% in 2011 this also is above the accepted dropout rate of 10%. The DPT3 coverage for Kadoma City in 2011 was 83% which is also below the district and national target of 90%. The OPV1, Pneumococcal 1, and Pentavalent 1 coverage at 6 weeks was 74% and for OPV2, Pneumococcal 2, and Pentavalent 2 was 84% at 10 weeks. The coverage for OPV3, Pentavalent 3 and Pneumococcal 3 was 74% at 14 weeks for Kadoma City. Clinics such as Rimuka Family Child Health, Chemukute and Waverly had immunization coverage of less than 90% district target for all the antigens at 6, 10 and 14 weeks. Ngezi clinic had the least coverage of all the antigens with average immunization coverage of 73%.

There has been little research in Zimbabwe on the effect of SMS on improving immunization coverage. Low immunization coverage is normally associated with outbreaks of vaccine preventable diseases hence the need to improve the coverage. Kadoma City needs innovative strategies to improve immunization coverage so that it can achieve the district target of 90%. Failure to improve the immunization coverage will reverse the gains towards achieving Millennium Development Goal4 (MDG 4) by 2015. The use of short message services as an intervention has been shown to improve utilization health care services in some settings.

It is against this background that we carried out a Randomized Control Trial (RCT) to evaluate the use of SMS in encouraging parents to bring their children for immunizations. This study will enhance current efforts where health education has been strengthened after engaging the services of health promotion officers’ in-order to improve immunization coverage. The objective of the study was to measure the effectiveness of using short message services on immunization coverage in Kadoma urban. The study was carried in-order to find out if there is no difference on the immunisation coverage among those receiving short message reminders and routine immunisation health education and those receiving routine immunisation health educations only.

## Methods

A Randomized Control Trial was conducted at Kadoma City Clinics in Mashonaland West province of Zimbabwe namely Rimuka, Waverley Chemukute and Kadoma General Hospital. Woman or caregiver was recruited into the study soon after delivery or during the 3^rd^ and 7^th^ day visits after delivery of the baby. Eligible respondent must have a cell phone and a resident of Kadoma city. The minimum sample size in the control group and intervention group was 138 each, considering a dropout rate of 10%; the minimum respondents to be recruited into the study were 304 respondents.

The study participants were allocated into the intervention and the non-intervention arms. At study initiation study participants were assigned by computer generated random numbers to 1 of 2 groups: no short message service reminder and routine health education and those receiving short message service reminders and health education. Study participants were followed up for 14 weeks. The mothers were followed up by phoning them using the mobile numbers provided and also comparing the details provided with those in the clinic immunization registers. The recruitment of study participants started on the 1^st^ of January 2013 and they were followed up until the 31^st^ of August 2013.

In the intervention group, the mother or caregivers received the routine health education and also received automatic messages indicating the next appointment date on three occasions. In the non intervention group, the mothers or caregivers received the routine health education and were informed about their next scheduled visit. The first message was sent 7 days before the due date for the immunization as a reminder. The second message was sent 3 days before the due date. The last message was sent a day before immunization appointment date. The messages were sent for the 6th, 10th and 14 weeks appointments.

The translated messages were as follows; A week before appointment date: − “*Immunization protects your child against killer diseases such as polio, whooping cough, diphtheria, measles, pneumonia and tuberculosis. You are reminded that the vaccination appointment will be due in 7 days time from today*.” Three days before appointment: − “*You are reminded that the vaccination appointment will be due in 3 days from today*.” A day before appointment: − “*Your vaccination appointment is due tomorrow, visit the nearest clinic*”.

Immunization delay was defined as the number of days after the immunization appointment day to the day the child receives the scheduled vaccine.

The primary outcome measure was receipt of scheduled vaccines at 6, 10 and 14 weeks. The secondary outcome measures were; delay in immunization appointment, age of child when immunized, costs, and willingness to receive SMS. Data were entered and analysed using Epi Info 7™ (CDC 2012).

Permission to carry out the study was obtained from Kadoma City Council; Joint Parirenyatwa Hospital and College of Health Sciences Research Ethics Committee OHRP IRB Number IORG 00008914 (JREC Ref 31/13) and; the Medical Research Council of Zimbabwe (MRCZ/B/492). Informed written consent was obtained from the respondents.

## Results

### Study respondents

**Figure 1:**
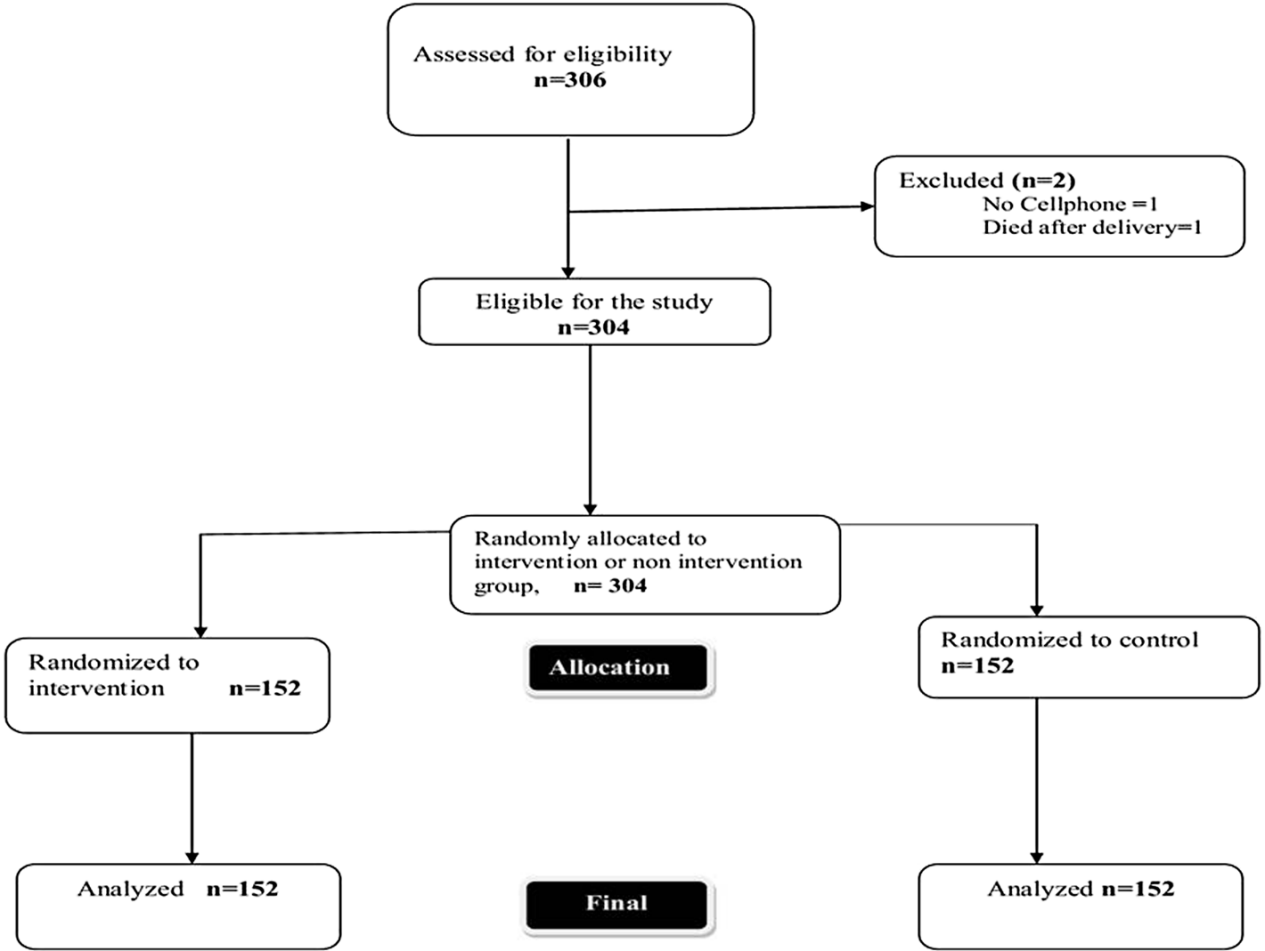
**Flow of study respondents Kadoma City, Zimbabwe, 2013.**

A total of 306 prospective respondents were assessed for eligibility and 304 were recruited into the study. One participant was excluded because she did not have a cell phone and one was not included because the mother died soon after delivery. A total of 152 participants were assigned to the intervention group and they received the short message service as immunization reminders. A further 152 were assigned into the non intervention group and did not receive the short message reminders. A total of 1377 messages were sent to the intervention group at 6, 10 and 14 weeks and all were delivered to the study respondents. All the respondents in both intervention and non intervention groups were followed up at 14 weeks. The flow of respondents is shown in Figure 1. Majority of the respondents were married, attained secondary level and were urban dwellers both in the intervention and non-intervention group. Table 1 summarizes the demographic characteristics of the participants.

**Table 1:** **Demographic characteristics of participants, Kadoma City, Zimbabwe, 2013**

**Variable**	**Intervention group n = 152(%)**	**No Intervention group n = 152(%)**
**Marital status**		
Married	139(91)	150(98)
Single	12(8)	2(1)
Separated	1(1)	1(1)
**Place of residence**		
Farm	8(5)	13(9)
Mine	8(5)	12(8)
Rural	8(5)	7(5)
Urban	128(84)	120(79)
**Highest level of education**		
No Education	1(1)	2(1)
Primary	15(10)	10(7)
Secondary	134(88)	132(87)
Tertiary	2(1)	8(5)
**Employment status**		
Full-time	19(13)	24(16)
Part-time	11(7)	15(10)
Unemployed	121(80)	113(75)
**Religion**		
Apostolic	40(26)	51(33)
Evangelical	59(39)	53(35)
Protestant	48(32)	44(29)
Islam	2(1)	2(1)
Traditional	3(2)	2(1)
**Median age (Years)**	26(Q_1_ = 21;Q_3_ = 30)	27(Q_1_ = 23;Q_3_ = 32)

### Immunization coverage at 6, 10 and 14 weeks

**Figure 2:**
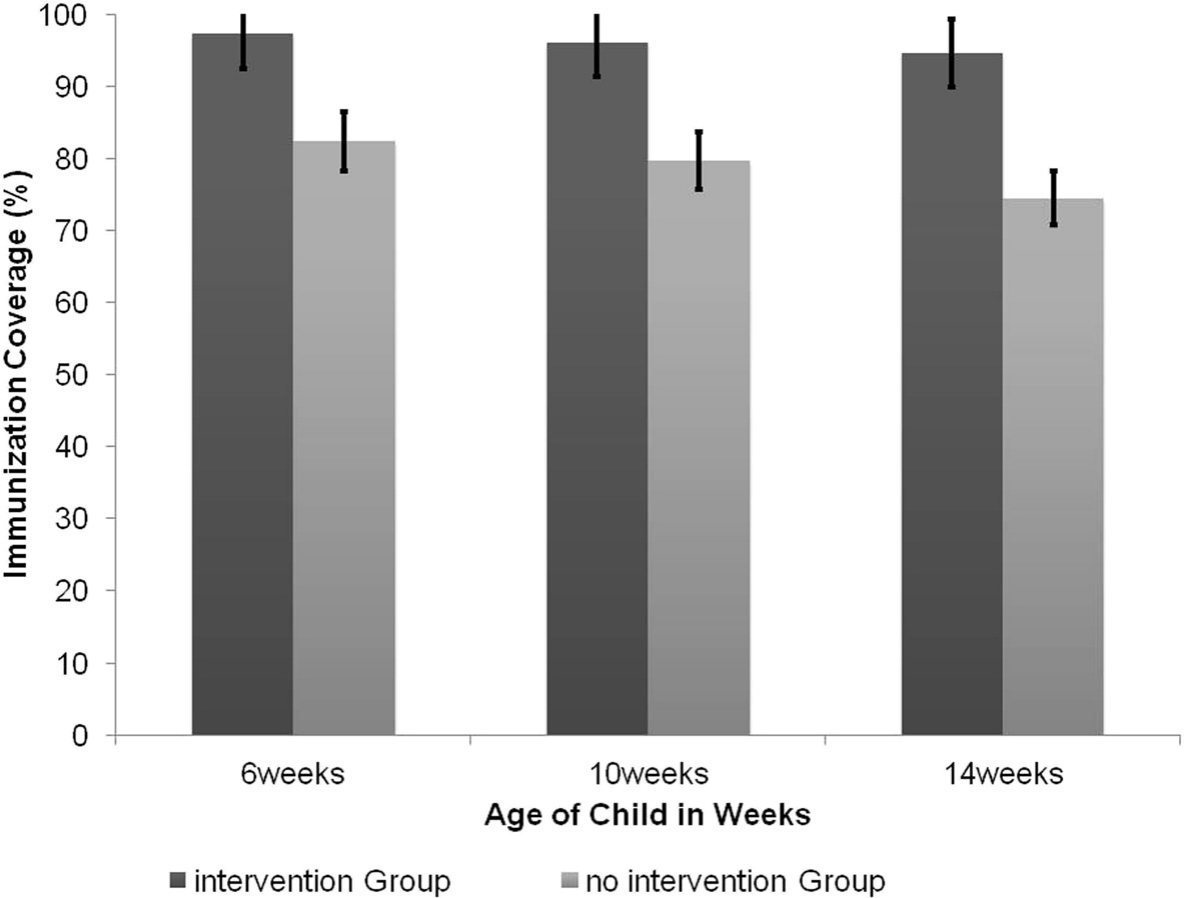
**Immunization coverage at 6, 10 and 14 weeks, Kadoma City, Zimbabwe, 2013.**

At 6 weeks OPV1, Penta1 and PCV1the immunization coverage in the intervention group was 97% and in the non intervention group was 82%. (p < 0.001). At 10 weeks the immunization coverage for OPV2, Penta2 and PCV2 was 96% in the intervention group and 80% in the non intervention (p < 0.001). Immunization coverage at 14 weeks for OPV3, Penta3 and PCV3 was 95% in the intervention group and 75% in the non intervention group (p < 0.001). Figure 2 summarizes the immunization coverage at 6, 10 and 14 weeks.

### Delay in immunization appointment

The proportion of those who did not delay in receiving OPV1, Penta1 and PCV1 at 6 weeks was 93% in the intervention group and 24% in the non intervention group. Among those in the non intervention group 4(2%) of babies were immunized before their appointments were due. The median delay in receiving OPV1, Penta1 and PCV1 in the intervention group was 0 days (Q_1_ = 0; Q_3_ = 0) whilst in the non intervention group the median delay was 2 days (Q_1_ = 0; Q_3_ = 5).

At 10 weeks the proportion of those who did not delay immunization in the intervention group was 87% and 17% in the non intervention group. The median delay in receiving the vaccines at 10 weeks was 0 days in the intervention group whilst in the control group it was 5 days (Q_1_ = 2; Q_3_ = 9).

The proportion of those who did not delay in receiving OPV3, Penta3 and PCV3 at 14 weeks was 82% in the intervention group and 8% in the non intervention group. The median delay in the intervention group was 0 days (Q_1_ = 0; Q_3_ = 0) whilst the median delay in the control group was 10 days (Q_1_ = 6; Q_3_ = 17). Those who delayed by more than 14 days was 30% in the control group.

### Age of child when immunized

The median age when OPV1, Penta1 and PCV were given is 41 days (Q_1_ = 41; Q_3_ = 41) in the intervention group and 44 days (Q_1_ = 42; Q_3_ = 46) in the non intervention group. In the intervention group 96% of the children were immunized when they were 41 days and 42 days old. In the non intervention group 34% were immunized at the exact age.

The median age of children who were immunized for OPV2, Penta2 and PCV2 were 70 days (Q_1_ = 70; Q_3_ = 71) in the intervention group and 75 days (Q_1_ = 72; Q_3_ = 79) in the non intervention group. Those that were immunized at the correct of 70 days were 69% in the intervention group and 12% in the non intervention group. The median age of the children when OPV3, Penta3 and PCV3 were given were 97 days (Q_1_ = 97; Q_3_ = 98) in the intervention group and 107 days (Q_1_ = 103; Q_3_ = 116) in the non intervention group. Those that were immunized at the correct age of 98 days were 90% in the intervention group and 12% in the non intervention group.

### Association between receiving short message services and receiving the targeted antigens

Respondents who received short message reminders at 6 weeks were 1.2 times more likely to have their children given OPV1, Penta1 and PCV1 at 6 weeks than those who did not receive short message service reminders (p < 0.001). The risk difference for those who received short message services and those who did not receive short message services was 15% (95% CI: 8.5-21.6). About 15% of the children immunized in the intervention group is attributed to SMS reminders and could not have been immunized if SMS reminders were not used at 6 weeks.

The respondents who received short message services at 10 weeks were 1.2 times more likely to have their children given OPV2, Penta2 and PCV2 than those in the non intervention group (p < 0.001). The risk difference for those who received short message reminders and those who did not receive short message reminders was 16.3% (95% CI: 9.2-23.4). About 16% of the children immunized in the intervention group is attributed to SMS reminders and could not have been immunized if SMS reminders were not used at 10 weeks.

The respondents who received short message services reminders were 1.3 times more likely to have their children immunized at 14 weeks than those who did not receive the short messages reminders (p < 0.001). The risk difference for those who received short message services reminders than those in the non intervention group was 16.3% (95% CI: 12.5-28.0). About 16% of the children immunized in the intervention group is attributed to SMS reminders and could not have been immunized if SMS reminders were not used at 14 weeks.

### Association between receiving short message services and delay in receiving the targeted antigens

The respondents who received short message services reminders were 89% less likely to delay in having their children immunized at 6 weeks than those who were in the control group (p < 0.001). The respondents who received short message services reminders were 81% less likely to delay in having their children immunized at 10 weeks than those who did not receive short message services (p < 0.001). The respondents who received short message reminders were 75% less likely to delay than those who did not receive the messages (p < 0.001).

### Costs associated with short message services for childhood immunization in Kadoma

A total of 1368 short messages were send to study participants in the intervention group and 42 messages were send to the researcher indicating those that are due for follow up. Messages to the study participants costed US$57.46, and the cost of messages to the researcher was US$1.76, giving a total cost of US$59.22 for all the messages that were send for the study. Capturing of data before sending short message reminders required about 5 minutes and this will translate to US$0.33 per message for the human resource needed.

### Willingness to receive short message service reminders

All the respondents in the intervention and non-intervention group were all willing to receive short message services and the preferred language was Shona (Table 2). Majority of the respondents preferred to be reminded a day before appointment. In the intervention group, 65% of the respondents preferred a day before appointment and in the non-intervention group it was 67%. In the intervention group 93% of the respondents perceive that the use of short message services is very beneficial compared to 97% in the non-intervention group.

**Table 2:** **Respondents’ attitudes towards SMS reminders for childhood immunization appointments, Kadoma City, Zimbabwe, 2013**

**Variable**	**Intervention**	**Control**	**p-value**
	**n = 152(%)**	**n = 152(%)**	
**Willing to receive SMS reminders about child’s immunization- Yes**	152(100)	152(100)	-
**Preferred language for Immunization SMS reminder-** Shona	152(100)	152(100)	
**Preferred time of SMS reminder**			
A day before appointment	98(64.5)	102(67.1)	0.8
Three days before appointment	42(27.6)	47(30.9)	0.6
A week before appointment	6(3.9)	1(0.7)	0.1
Other	6(3.9)	2(1.3)	0.1
**Perception of benefit expected to be received via SMS**			
Very beneficial	141(92.8)	148(97.4)	0.1
Somewhat beneficial	2(1.3)	1(0.7)	0.6
Not beneficial	6(3.9)	1(0.7)	0.1
Indifferent	3(2.0)	2(1.3)	0.7

## Discussion

In this study there was no significant difference in the baseline demographic characteristic of those in the intervention and control groups. This could be indicating that randomization was well achieved. All the respondents who were enrolled into the study at the beginning of the study were all followed up and none were lost to follow up. The comparison is thus optimal to estimate the true benefits of the use of short message reminders because all the study participants who were randomized were included in the analysis. Control of the unknown confounders is likely to have been achieved in this study since this is likely to be distributed equally during randomization.

The immunization coverage in this study at 6, 10 and 14 weeks was significantly higher in the intervention group than in the non-intervention group (p < 0.001). The significant difference in the immunization coverage can be attributed to the use of immunization short message reminders. The findings in this study are similar to those reported by Eugene F *at el*. (1995) who evaluated the effectiveness of reminders in increasing kept appointment rates on immunization in a public health setting. In the study by Eugene those who were receiving reminders had significantly high kept appointments. However, unlike our study were SMS reminders were used Eugene used computer generated telephone reminders [14-16].

In this study the proportion of those who did not delay in receiving antigens at 6, 10 and 14 weeks was significantly small compared to those in the non intervention group (p < 0.001). The use of short message reminders could have caused the women to bring their children on time compared to those in the non-intervention. Prasad and Anand (2012) in a Randomized Control Trial conducted in the United Kingdom reported that there was an overall increase in fulfilling appointment of 79% in the intervention and 34% in the non intervention. The difference between our study and Prasad’s study was the broader outcome measure that is attending on the appointment day.

The median age of the child in this study when antigens were given at 6, 10 and 14 weeks were significantly different in the intervention group and in the non intervention group. Those in the intervention group were being immunized at the correct age. In the non intervention group, they were being immunized when they had already passed their immunization age. Failure to immunize the children at their correct ages will expose children to some of these vaccine preventable conditions [6,17-21].

Messages to the study participants costed US$57.46, and the cost of messages for the entire immunization schedule of one child upto 18 months it will be including human resource for capturing data was US$0.99, if the child is receiving 3 messages prior to the due date. However if only one message will be send to the child the cost will be US$0.21 per child for the entire immunization programme. Considering the benefits of timely immunization in fighting child morbidity and mortality the cost will be worthwhile. The under one population in Kadoma is approximately 2500, and the approximate cost for sending short message reminders will be US$2500 per year provided they are sending 3 messages per every immunization visit. Considering that they are currently more than this amount when doing immunization mobilization, the use of short message reminders will be affordable to Kadoma City [18,22-28].

In this study all the respondents in the intervention and non-intervention group were all willing to receive short message services and the preferred language was the local language Shona. If all the respondents are willing to receive messages this will be good because if they were not willing it was not going to be possible to use the short message reminders to improve immunization coverage. In this study all the respondents preferred local language Shona, so this will allow programming easy because only one standard message will be used. This is in contrast with study findings in Nigeria by Balogun *et al.* in 2012 who found out that mother preferred short service messages in English language than their local language [15,17,29].

In this study majority of the respondents preferred to be reminded a day before appointment and they perceive that the use of short message services is very beneficial. This is also similar to findings again by Balogun *et al.* in 2012 in Nigeria on the willingness to receive text message reminders on childhood immunization among women attending a tertiary hospital in Lagos found that the majority of the respondents were willing to receive SMS immunization reminders. The mothers in the Nigerian study had a positive attitude towards reminders and appreciated the benefit it would have to them and their children [15,30-34].

## Conclusion

Immunization coverage was high in the intervention group than in the non intervention. The overall increase may be attributed to the use of SMS reminders in this study. The use of short message service reminders was associated with no immunization delay. All the respondents were willing to receive immunization SMS reminders and they perceive them as very beneficial. The preferred language for short message service immunization reminders is Shona. The cost of short message service reminders for the immunization schedules upto 18 months is US$0.99 per child if receiving 3 messages for each visit. Adoption of SMS use in Kadoma City will improve immunization coverage.
